# Blood glucose levels in patients with metastatic renal cell carcinoma treated with sunitinib

**DOI:** 10.1038/sj.bjc.6604709

**Published:** 2008-10-07

**Authors:** B Billemont, J Medioni, L Taillade, D Helley, J B Meric, O Rixe, S Oudard

**Affiliations:** 1 Department of Medical Oncology, Pitie-Salpetriere Hospital, Paris, France; 2 Department of Medical Oncology, Georges Pompidou Hospital, Paris, France; 3 Department of Biology, Georges Pompidou Hospital, Paris, France

**Keywords:** renal cell carcinoma, sunitinib, diabetes, glucose level, tyrosine-kinase inhibitor, VEGF

## Abstract

Sunitinib, a multitargeted tyrosine-kinase inhibitor, extends survival of patients with metastatic renal cell carcinoma (mRCC) and gastrointestinal stromal tumours. Between October 2005 and March 2007, we retrospectively reviewed blood glucose level variations associated with sunitinib therapy in patients treated for mRCC. Nineteen of the patients had type II diabetes. All 19 patients had a decrease in blood glucose level (mean 1.77 mmol l^−1^) after 4 weeks of treatment. This was followed by re-elevation in the 2-week rest period. After two cycles of sunitinib administration, two patients had stopped blood glucose-lowering drugs whereas five other patients had normalised their blood glucose level. On the basis of pre-clinical data, we hypothesise that several mechanisms could be involved in this process, such as capillary regression of pancreatic islets, IGF-1 modulation through HIF1-*α* or NF-*κ*B activation. In addition, a decrease of glucose uptake in the context of concomitant gastrointestinal toxicity cannot be excluded. Glycaemic control should be carefully evaluated in diabetic patients treated with sunitinib, and routine monitoring is warranted.

Sunitinib (sunitinib malate; SU11248; SUTENT®; Pfizer Inc., New York, NY, USA) is an orally bioavailable, oxindole, multi-targeted tyrosine kinase inhibitor with antitumour and antiangiogenic activities. Sunitinib has been identified in both biochemical and cellular assays (Faivre *et al*, 2007) as a potent inhibitor of VEGFRs (types 1–3), PDGFR (*α* and *β*), as well as FLT3, Kit (stem-cell factor (SCF) receptor), colony-stimulating factor type I (CSF-1R) and glial cell-line-derived neurotrophic factor receptor (RET).

Antitumour activity in metastatic renal clear cell carcinoma (mRCC) has been demonstrated in phase II (Motzer *et al*, 2006) and in phase III studies (Motzer *et al*, 2007). In the international randomised phase III trial (Motzer *et al*, 2007), the efficacy and safety of sunitinib as compared with IFN-*α* in first-line treatment of patients with mRCC were evaluated. Median progression-free survival was 11 months (95% CI: 8.14) for sunitinib *vs* 5 months for IFN-*α* (95% CI: 4.6), corresponding to a hazard ratio of 0.42 (95% CI: 0.32–0.54) (*P*<0.001). The objective response rate, by a third-party independent review was 31% for sunitinib *vs* 6% for the IFN-*α* (*P*<0.001). In all, 632 patients (85%) are alive, with 49 deaths on the sunitinib arm and 65 deaths on the IFN-*α* arm. On the basis of this large phase III study, sunitinib became one of the standard therapies for the first-line treatment of mRCC.

The main toxicities reported in the phase II and III trials with sunitinib were fatigue, hand/foot syndrome, intestinal toxicity (including mucositis and diarrhoea) and hypertension. Eight percent withdrew from the phase III study due to adverse events on the sunitinib arm *vs* 13% on the IFN-*α* arm (Motzer *et al*, 2007). In addition to the side effects described in these publications, atypical toxicities, such as hypothyroidism (Rini *et al*, 2007) or vitamin deficiencies (Gillessen *et al*, 2007) have been reported.

Recently, Templeton *et al* (2008) reported an interesting clinical case of remission of type I diabetes after sunitinib treatment. In this report, we describe our investigation of blood glucose level variations in a large cohort of patients treated with sunitinib.

## Patients and methods

Between October 2005 and March 2007, 200 patients with mRCC, included in the phase III study or in an expanded access programme, have been followed at the Pitie-Salpetriere and George-Pompidou hospitals. All patients signed a written informed consent form that was approved by external review boards and were treated with sunitinib at the initial dose of 50 mg daily (50 mg given daily, 4 weeks on, 2 weeks off). The medical records of patients with mRCC treated in our institutions with sunitinib were retrospectively reviewed for glucose blood level alterations and use of blood glucose-lowering drugs. The main inclusion criteria for this retrospective study were age 18 or older, histologically or cytologically confirmed mRCC, adequate hepatic, renal and bone marrow function, and repeated fasting glucose blood analysis at baseline and on days 1 and 28 of each cycle. All patients with type I or 2 diabetes, defined as case patients, were compared with control patients with mRCC. Control patients were defined consistently normoglycaemic at baseline and during sunitinib treatment. The longitudinal analysis of blood glucose level variation was performed with the non-parametric Wilcoxon test. Patients were considered as their own control in paired analysis.

## Results

In our analysis, 19 patients had type II diabetes. Five patients out of 19 had pancreatic metastases. The mean baseline blood glucose level for all 19 was 8.26 mmol l^−1^ (range 4.05–13.80). These 19 patients were treated with oral antihyperglycaemic medications including metformin, glibenclamide and gliclazide. Two patients were insulin dependent. The demographic characteristics of patients treated are presented in Table 1.

**Figure 1:**
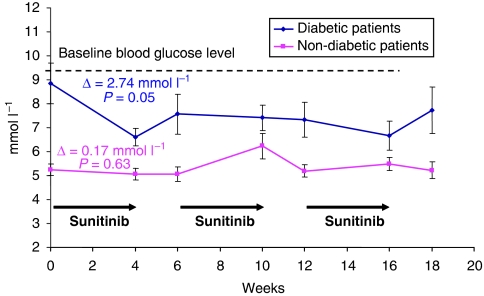
Mean blood glucose levels in 19 type II diabetic patients and 9 non-diabetic patients during sunitinib treatment for mRCC.

After 4 weeks of treatment, all 19 patients had a decrease of blood glucose level (mean 1.77 mmol l^−1^; *P*=0.05), and this was followed by an increase of blood glucose level during the rest period (mean 0.93 mmol l^−1^) (Figure 1). After two cycles of sunitinib (12 weeks), two patients were able to stop their antihyperglycaemic treatment during the treatment phase and reinitiated their medication during the sunitinib rest period. Five other patients have normalised their blood glucose level. No severe episode of hypoglycaemia has been reported.

By comparison, blood glucose level from nine non-diabetic ‘control’ patients (Figure 1) were analysed, and a nonsignificant decrease of mean level was observed from 5.89 to 5.26 mmol l^−1^ (*P*=0.79). The trend over the time of blood glucose levels seems to be different for non-diabetic patients with no variation during sunitinib therapy or in the 2 weeks off treatment. In a global analysis of all 28 patients treated with 4 weeks of sunitinib, the blood glucose level decreased from 7.59 to 6.03 mmol l^−1^ (*P*=0.04).

## Discussion

Type II diabetes mellitus is an extremely complex disorder involving insulin resistance and *β*-cell dysfunction. Insulin receptors and IG1 receptors activate a number of post-receptor cascades including Irs, Sgk or Akt-2 protein kinases inducing protein synthesis, antilipolysis and cell survival (Leroith and Accili, 2008). The exact mechanism involved in our observation remains to be elucidated but several hypotheses can be elaborated.

First, Kamba *et al* (2006) have reported similar observations in mice models using AG-013736, which is also a tyrosine kinase inhibitor of VEGFR-1, -2 and -3. In this animal model, a 21-day pre-treatment with AG-013736 improved blood glucose handling. According to the authors, this phenomenon could be partially related to significant quantitative and qualitative capillary regression in pancreatic islets. Interestingly, this VEGF-dependant phenomenon was reversible after cessation of the treatment. Second, IGF-1 regulates VEGF expression through HIF1-*α* (Carroll and Ashcroft, 2006). We could hypothesise that sunitinib treatment could interfere with the IGF-1 pathway, having a subsequent impact on insulin resistance. To investigate this hypothesis, IGF-1 serum levels were monitored in five patients in our diabetic cohort. Although a decrease was observed at week 4, this was not significant (data not shown, *P*=0.06). Third, amelioration of diabetes by imatinib (Gleevec®) has been reported in animal models (Hagerkvist *et al*, 2007) and in human (Veneri *et al*, 2005). This unexpected effect was related to the protective effect of imatinib on *β* cells by an antiapoptotic action through NF-*κ*B activation. However, an effect of sunitinib on NF-*κ*B modulation has yet not been reported. Modulation of the IGF pathway has also been implicated in exacerbation of hypoglycaemia by imatinib in patients with non-islet-cell tumours (Hamberg *et al*, 2006).

A decrease of glucose uptake in a context of concomitant gastrointestinal toxicity cannot be excluded. Anorexia and intestinal toxicity (Motzer *et al*, 2007) have been reported in the phase III study and oral glucose uptake could consequently be reduced. In addition, direct intestinal toxicity of sunitinib has been reported, and this could induce vitamin malabsorption (Gillessen *et al*, 2007). However, the hypothesis of altered glucose transport is not confirmed, as glucose handling was not modified in the animal model after an intravenous compared with an oral glucose challenge after AG-013736 treatment (Kamba *et al*, 2006). Finally, drug–drug interaction (between sunitinib and blood glucose-lowering drugs) could be advanced as an additional basis for our findings. A pharmacokinetic analysis to explore the relationship between sunitinib and blood sugar levels deserves further investigation.

Our data suggest that sunitinib lowers the blood glucose level. The indications for and the dose of blood glucose-lowering drugs should be evaluated during both the active treatment period and the rest period. To avoid severe hypoglycaemia, oral blood glucose-lowering drugs should be carefully monitored in this context. A potential relationship with sunitinib efficacy should be addressed in a large prospective analysis.

## Figures and Tables

**Table 1:** Baseline characteristics of the 28 patients with metastatic renal cell carcinoma

**Characteristics**	**Type 2 diabetic *N*=19**	**Non-diabetic *N*=9**
Sex		
Male/female	18/1	8/1
		
*Age (years)*		
Mean (range)	62 (52–74)	57 (42–77)
PS ⩽1	18	8
		
*Diagnostic age*		
Mean (range)	61 (47–65)	53.7 (39–73)
		
*Metastatic age*		
Mean (range)	61 (50–72)	56 (42–77)
		
*Histology*		
Clear cell	19	8
Chromophobe	0	1
Nephrectomy	19	7
Primary tumour	0	2
		
*Pre-treatment*		
⩽2 lines	10	4
INF	16	9
IL-2	1	2
INF+IL-2	2	0
Hormonotherapy	2	1
Radiotherapy	2	1
Antiangiogenic agents	8	9
Chemotherapy	2	1
		
*Metastasis site*		
Liver	5	5
Lung	16	7
Pancreas	5	1
>2 sites	10	4
		
*Diabetes treatment*		
Insulin	2	0
Metformin	4	0
Glibenclamide	0	0
Glicazide	10	0
Repaglinide	1	0
Benfluorex	1	0
Biguanide+glitazone	1	0
Pioglitazone	1	0
		
*IGF-1*	5 patients	—
Baseline, median–mean (range)	56.4–59.3 (46.3–82.4)	—
Week 4, median–mean (range)	62–119.5 (29–64.1)	—

IGF-1=insulin growth factor; IL-2=interleukin-2; INF=interferon.
